# The Critical Role of DNA Extraction for Detection of Mycobacteria in Tissues

**DOI:** 10.1371/journal.pone.0078749

**Published:** 2013-10-23

**Authors:** Nicolas Radomski, Louis Kreitmann, Fiona McIntosh, Marcel A. Behr

**Affiliations:** 1 Research Institute of the McGill University Health Centre, Montreal, Québec, Canada; 2 Department of Medicine, McGill University, Montreal, Québec, Canada; 3 McGill International TB Centre, Montreal, Québec, Canada; University of Illinois at Chicago College of Medicine, United States of America

## Abstract

**Background:**

Nucleic acid-based methods offer promise for both targeted and exploratory investigations of microbes in tissue samples. As the starting material for such studies is a mixture of host and microbial DNA, we have critically evaluated the DNA extraction step to determine the quantitative and qualitative parameters that permit faithful molecular detection of mycobacteria in infected tissue. Specifically, we assessed: 1) tissue disruption procedures; 2) DNA extraction protocols; and 3) inhibition of bacterial PCR by host DNA.

**Principal Findings:**

Regarding DNA extraction, we found that 1) grinding was not necessary if bead-beating is done, 2) the reference mycobacterial DNA extraction method recovered more pure DNA than commercial spin column kits, 3) lysozyme digestion of 1 hour was sufficient, and 4) repeated steps of phenol:chloroform:isoamyl alcohol offered minimal gain in DNA quality. By artificially mixing mycobacterial DNA with DNA extracted from uninfected mice, we found that bacterial real-time quantitative PCR was only reliable when the quantity of host DNA was < 3 µg in a final volume of 25 µl and the quality was high (260/280 nm ratio = 1.89±0.08). Findings from spiked DNA studies were confirmed using DNA extracted from mice infected with different intracellular pathogens (*M. tuberculosis*, *M. avium* subsp. *paratuberculosis*).

**Conclusions:**

Our findings point to the most appropriate methods for extracting DNA from tissue samples for the purpose of detecting and quantifying mycobacteria. These data also inform on the limits of detection for two mycobacterial species and indicate that increasing the sample mass to improve analytic sensitivity comes at the cost of inhibition of PCR by host DNA.

## Introduction

Molecular techniques offer culture-independent ways for diagnostic labs to test tissue samples for specified organisms or for researchers to investigate the presence of unknown pathogens. Regardless of the nucleic acid-based technique employed, when beginning with a biopsy sample, the starting material for the molecular assay is a mix of host and microbial DNA [1]. As there are currently no standards for bacterial DNA extraction from tissue samples, a variety of different methods has been employed by different groups. Indeed, a search of the published literature reveals that some studies used kits that enrich nuclei, where bacteria are not located [2], while others have used in-house DNA extraction methods [3-7], commercial kits [8], or both merged together [9,10].

In the absence of a standardized DNA extraction method, studies have attempted to control for the possibility of false-negative results by adding into the real-time PCR reaction [11] an internal amplification control (IAC) that targets a host gene [12], a plasmid [12,13], or exogenous bacterial DNA [14]. While these ‘spiked’ templates can test for PCR inhibition, the use of an IAC does not formally test for the capacity to detect the DNA of a microbe that is embedded within the sample. This issue is especially problematic for *Mycobacterium* spp., because a) DNA extraction is difficult even when mycobacterial cells are grown in pure culture, due to their complex cell wall [15-17], b) the bacteria cell is within a phagosome, within a host cell, within a granuloma, necessitating several steps of stripping away host contaminants, and c) infection can be associated with disease even with a low burden of bacteria (paucibacillary disease, e.g. tuberculoid leprosy).

To address this issue, we have evaluated different DNA extraction techniques, using two different mycobacterial species (*M. avium* subsp. *paratuberculosis* and *M. tuberculosis*) and both spiked samples and experimentally defined infections. Using quantitative real-time PCR, we assess both the limits of detection of mycobacteria in tissue and determine the effect of host DNA on the fidelity of the bacterial quantification.

## Methods

### Ethics Statement

Animal procedures were done in accordance with the guidelines established by The Canadian Council on Animal Care, and were approved by The McGill University Animal Care Committee [18].

### Bacteria and growth conditions

*M. avium* subsp. *paratuberculosis* (MAP) K-10 and *M. tuberculosis* (H37Rv and H37Ra) were grown in Middlebrook 7H9 (Becton, Dickinson and Company) with 1 μg/ml of mycobactin J (Allied Monitor Inc.) for MAP.

### Murine infections and harvesting of tissue

As previously described, C57BL/6 mice were infected with 10^8^ or 10^6^ MAP K-10 by intraperitoneal injection [19] or with 10^2^ *M. tuberculosis* H37Rv by aerosolization [20] using an appropriate equipment (Lovelace aerosol nebulizer, In-Tox products). In addition we also harvested organ samples from non-infected mice that were kept and processed in parallel as negative controls. At the indicated time, lungs and spleens (*M. tuberculosis*) or livers and spleens (MAP) were harvested by aseptic technique and weighed, to allow molecular quantification of infection and determination of bacterial burden per mg of tissue.

### DNA extraction protocol

Because DNA extraction kits have been reported to recover low amounts of pure mycobacterial DNA [21-24], we compared the most widely used DNA extraction procedure for mycobacterial DNA [3-6,15,17], first described by van Soolingen et al. [16] against two kit-based DNA extraction procedures using silica membranes: Invisorb Spin Tissue kit (Invitek) recently used for MAP DNA extraction [8] and PowerSoil DNA isolation kit (MoBio), used for the Human Microbiome Project [25]. For brevity, we will call the van Soolingen procedure the reference method.

Concerning comparison of DNA extraction procedures using mycobacterial cells as starting material, we prepared six replicated pellets of MAP and *M. tuberculosis* at 10 log_10_, 9 log_10_, and 8 log_10_ cells (total of 108 pellets for DNA extractions + 18 pellets for cell density check by culture plate). For each strain, a culture of 1,500 ml was prepared in Middlebrook 7H9 (1 μg/ml of mycobactin J for MAP) until to achieve an OD closed to 1.0. Using formula of correlation between cfu and OD (2.0×10^8^ cfu/ml for a culture at OD=1), cell density was estimated (1.96×10^8^ cfu/ml for MAP, and 1.99×10^8^ cfu/ml for *M. tuberculosis*), and a part of this culture was centrifuged 15 min at 3000×g in 250 ml tubes (1,174 ml for MAP and 1,157 ml for *M. tuberculosis*). After removal of supernatant, cell pellets were dissolved and merged in a final volume of 23 ml of media in order to achieve a theoretical cell density of 10^10^ cfu/ml. Then, 21 tubes of 1.5 ml containing 1 ml of this culture were prepared, centrifuged at 15 min at 3000×g, and supernatants were removed in order to obtain pellets at 10 log_10_ cells. Three of these pellets were used to check cell amount in triplicate assays by serial dilutions and plating on Middlebrook 7H10 (1 μg/ml of mycobactin J for MAP), and the other 18 pellets were used for comparison of DNA extraction procedure (6 replicated pellets for each method). The remaining culture at 10^10^ cfu/ml (2 ml) was diluted in 18 ml of media, and the same procedure was used to prepare 21 pellets at 9 log_10_ cells and check cell density by culture plate. Finally, the remaining culture at 10^9^ cfu/ml (2 ml) was diluted in 18 ml of media, and the same procedure was used to prepare 21 pellets at 8 log_10_ cells and check cell density by culture plate.

In studies that applied the reference method to biopsies, there were variations in several steps that might affect the quantity and quality of extracted DNA. We assessed the importance of host cell disruption (grinding and/or 1 vs. 2. vs. 3 bead beating steps) [3,4,26], the duration of lysozyme digestion (1h vs. 24h vs. 48h) [10,15,17], and the number of phenol:chloroform:isoamyl alcohol steps (1 vs. 2 washes) [3-6].

After DNA purification, equal volumes of isopropanol at +4°C (100%) were added to the upper phase previously transferred into a clean 1.5 ml tube, then tubes were slowly mixed by inversion and kept overnight at -20°C, before another centrifugation (15 min at 16000 ×g). Following removal of the supernatant, 1 ml of 70% ethanol at +4°C was added to the DNA pellets. These pellets were suspended by flicking the tubes, followed by inversion and centrifugation (15 min at 16000 ×g), then pellets were dried for 10 min (V-AQ mode, Vacufuge plus, Eppendorf) and 50 µl of nuclease-free water was added.

### Qualitative and quantitative assessment of extracted DNA

DNA purity was determined by the ratios 260 nm/280 nm (indicator of organic matter residues) and 260/230 nm (indicator of organic solvent residues) [27]. DNA amount was estimated at 260 nm by spectrophotometer (ND-1000 Spectrophotometer, NanoDrop).

**Figure 1:**
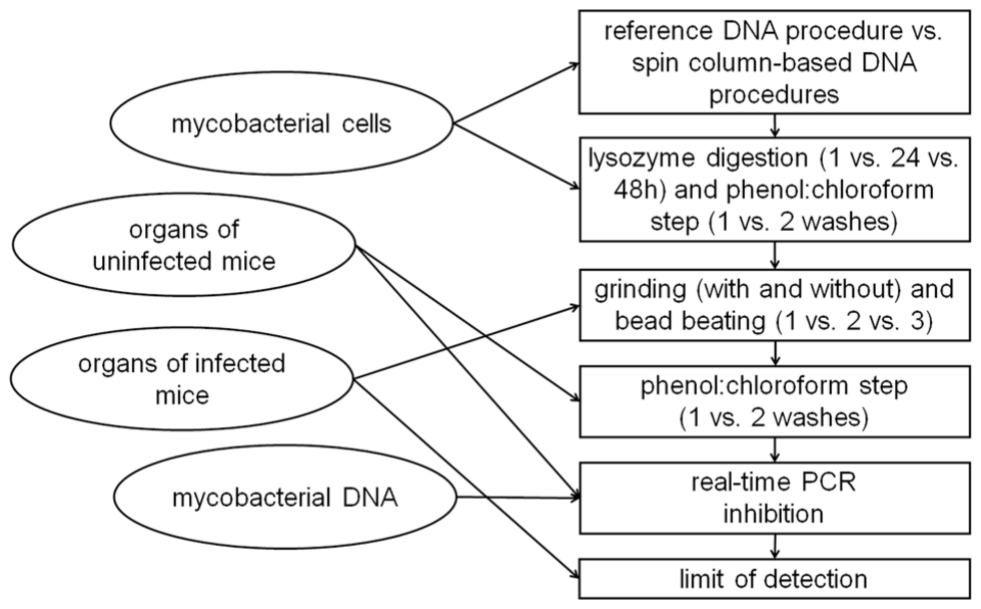
Flow diagram describing organization of samples (circles) and compared factors (rectangles) used for improvement of mycobacterial DNA detection by real-time PCR in tissues.

For the purposes of assessing PCR inhibition, from a total of 384 non-infected liver, spleen, and lung samples, the 36 most representative of the sample diversity, in term of DNA amount and DNA purity, were selected for each organ (Figure 1). Because the limit of quantification of the spectrophotometer is between 1 and 10 ng/µl, we omitted extracted DNA whose amount and purity could not be accurately estimated due to this limitation. In this case, since we used 5 µl of extracted DNA as template, the lower limit of reliable quantification was set at 50 ng (10 ng/µl × 5 µl).

### Real-time PCR assays

For the detection and quantification of MAP in organs, we used recently described protocol that amplifies MAP amplicon III f57 [8] from the MAP0865 gene (Table S1). For the detection of *M. tuberculosis* in organs, we tested for the sequence called ext-RD9 (Table S1) which is specific to *M. tuberculosis* complex (MTC) and serves in the differentiation of *M. tuberculosis* *sensu stricto* from *M. africanum, M. caprae* and *M. bovis* [28]. Host DNA was quantified targeting prostaglandin E receptor 2 (PTGER2) [29]. Quantification was performed by TaqMan real-time PCR, except for host genomes where we employed SYBR Green. More details on the real-time PCR assays are provided in Appendix S1. Ten-fold serial dilutions of all extracted DNA were performed from 10^-1^ to 10^-4^ before spectrophotometer measurements and real-time PCR quantification of each diluted sample.

### Data analysis

All statistical analyses were performed with JMP software version 10, and appropriate statistical tests are specified in results section for each comparison. More precisely, non-parametric tests are presented because distributions of the data are not Normal (Shapiro-Wilk test, p<0.001).

## Results and Discussion

### Comparison of procedures for mycobacteria DNA extraction in vitro

**Figure 2:**
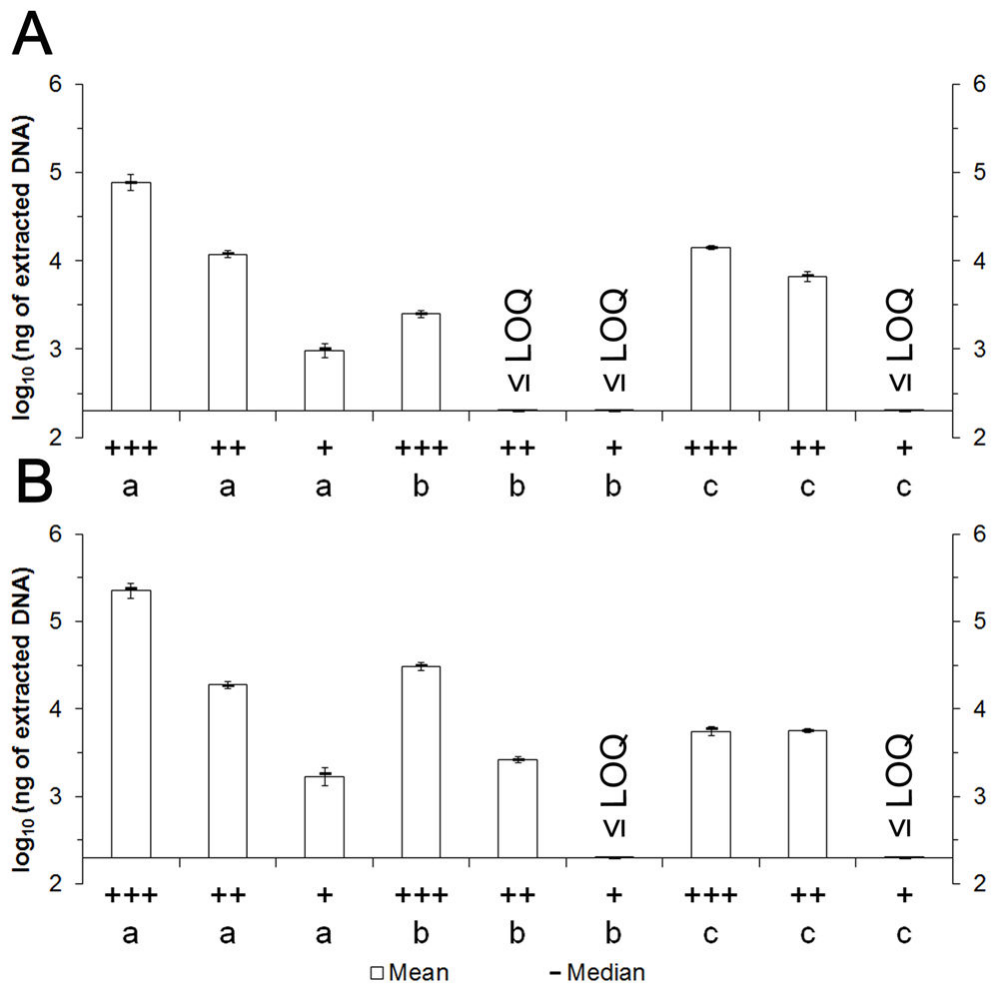
DNA amount measured at 260 nm after DNA extractions (a: van Soolingen procedure, b: Invisorb Spin Tissue Mini Kit from Invitek, c: PowerSoil DNA isolation Kit from MoBio) from big (+++: 10 log_10_ cells), medium (++: 9 log_10_ cells), and small (+: 8 log_10_ cells) pellets of MAP K-10 (A, n=54), and *M. tuberculosis* H37Ra (B, n=54) cells. Error bars are standard deviation. LOQ are limit of quantification of the spectrophotometer.

Cell densities of prepared pellets (10, 9, and 8 log_10_ cells) were estimated at 9.8±0.3 log_10_ cfu, 9.2±0.1 log_10_ cfu, and 8.1±0.2 log_10_ cfu for MAP, and at 9.7±0.2 log_10_ cfu, 9.1±0.3 log_10_ cfu, and 7.9±0.2 log_10_ cfu for *M. tuberculosis*. Using mycobacterial cell pellets, the quantity of extracted DNA from *M. avium* subsp. *paratuberculosis* (MAP) and *M. tuberculosis* was 10-100 times higher with the reference method than both kits (Wilcoxon/Kruskal-Wallis test: n=108, ddl=2, *p*<0.0001 for both). Specifically, from pellets at around 10 log_10_ cells (Figure 2), we obtained 78,400±16,060 ng for MAP (i.e. 10^4.9^ ng) and 230,367±42,718 ng for *M. tuberculosis* (i.e. 10^5.4^ ng) using the reference method. In comparison, the yield with Invitek was 2,510±223 ng for MAP (i.e. 10^3.4^ ng) and 30,437±3,051 ng for *M. tuberculosis* (i.e. 10^4.5^ ng) while the yield with MoBio was 14,200±687 ng for MAP (i.e. 10^4.2^ ng) and 5,620±633 ng for *M. tuberculosis* (i.e. 10^3.7^ ng). While mycobacterial cell density (10 log_10_, 9 log_10_, or 8 log_10_ per pellet) and DNA extraction method (reference vs. kits) were both significant predictors or the DNA yield (Figure 2), the mycobacterial species was not a predictor of the amount of extracted DNA (Wilcoxon/Kruskal-Wallis test: n=108, ddl=2, *p*=0.151). Since our goal was to determine the optimal protocol for detecting bacterial DNA upon liberation of bacterial cells from a tissue sample, we rejected these kit-based methods from further study.

### Optimization of DNA extraction method for MAP quantification in tissues

**Figure 3:**
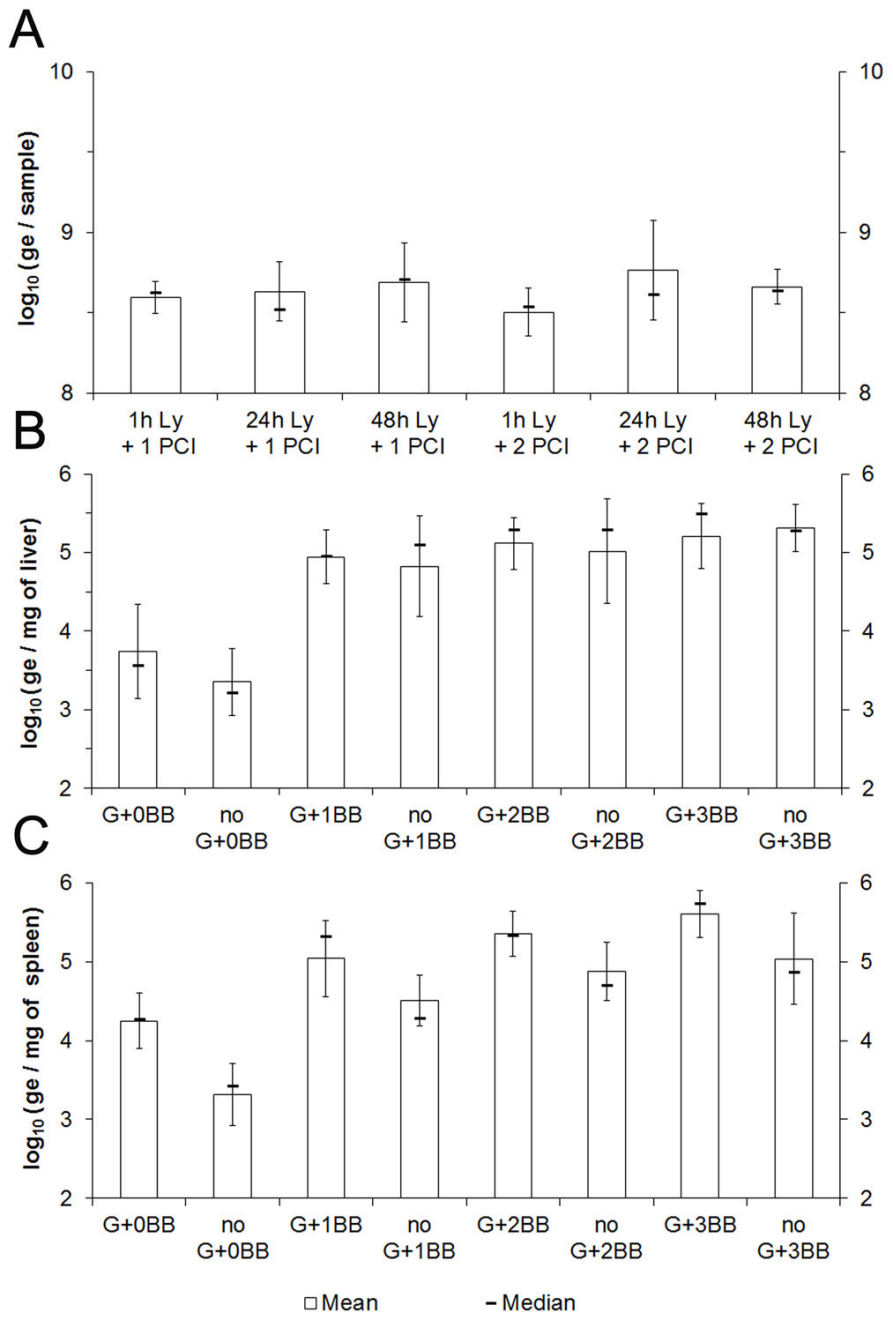
Comparison of MAP K-10 quantification methods in culture samples (A, n=28), or in liver (B, n=45) and spleen (C, n=45) samples from C57BL/6 mice: culture on 7H10-mycobactin *versus* TaqMan real-time PCR targeting MAP0865 gene, including 1 h, 24 h, or 48 h of lysozyme digestion (Ly), 1 or 2 phenol:chloroform:isoamyl purifications (PCI), no or 1 grinding step (G), and/or 0, 1, 2 or 3 steps of bead beating (BB) during DNA extraction procedure. Error bars are standard deviation.

When starting with live MAP grown in broth culture as the primary sample, DNA extraction procedures including 1 h, 24 h, or 48 h or lysozyme digestion, and/or, 1 or 2 phenol:chloroform:isoamyl (PCI) purification steps (Figure 3A) did not produce significant differences in yield (Generalized linear model: n=24, ddl=5, *F*=1.98, *p*=0.189). Using MAP-infected liver (Figure 3B) and spleen (Figure 3C) as samples, optimal disruption was achieved by bead beating; tissue grinding alone was neither sufficient nor necessary to achieve optimal levels of MAP DNA (Figure 3B and 3C). There was no benefit with greater than 1 step of bead beating. Based on these findings, we proceeded for subsequent experiments with: 1 bead beating step and 1h of lysozyme digestion.

### Limit of detection by real-time PCR of MAP and *M. tuberculosis* in vitro and in tissues

**Figure 4:**
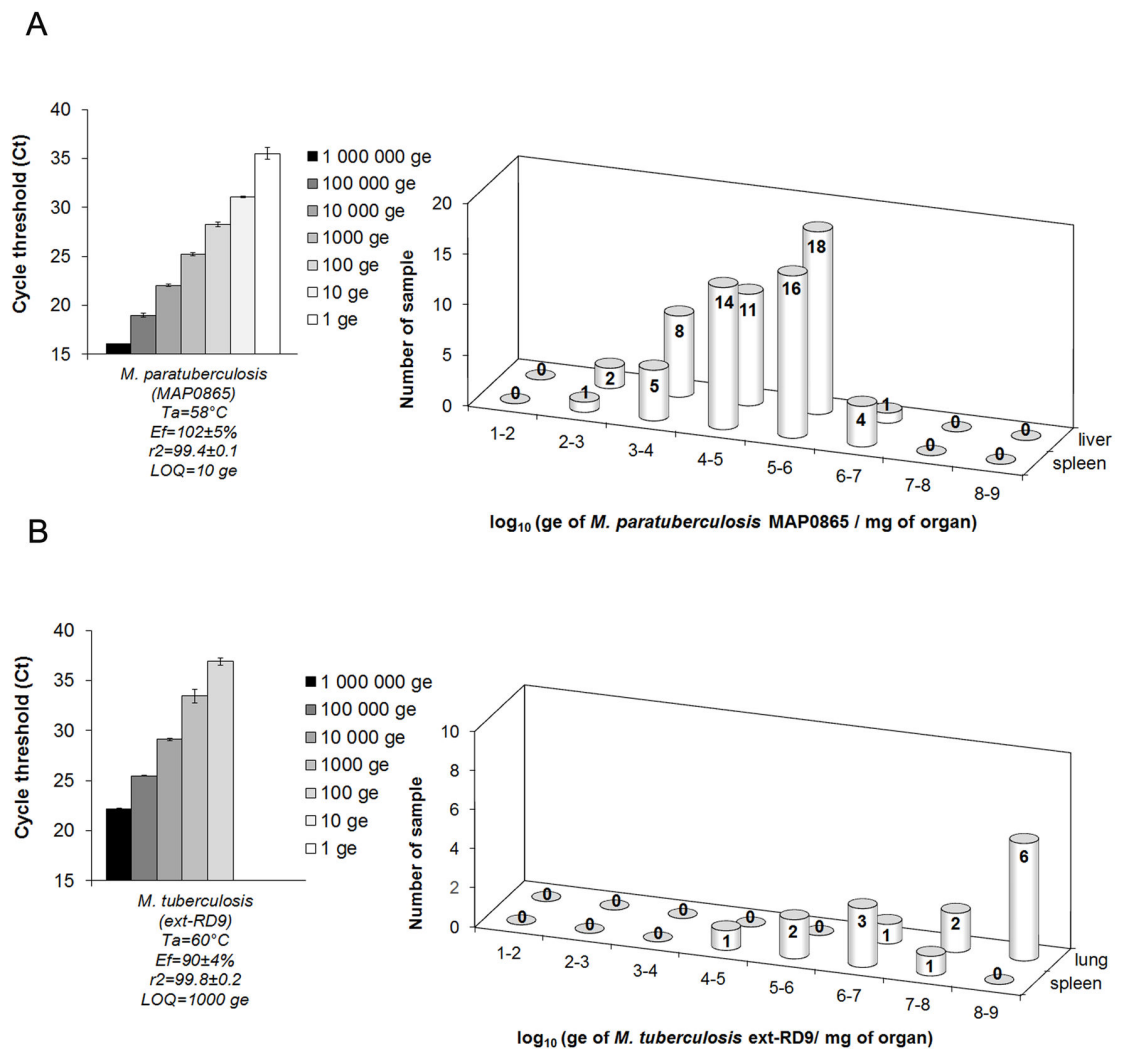
*in* *vitro* limit of quantification (on the left) estimated by reproducible TaqMan real-time PCR amplifications of MAP0865 (n=63) and ext-RD9 (n=21) for MAP K-10 (A) and *M. tuberculosis* H37Rv quantification (B), and limits of quantification in tissues (on the right) estimated by distribution analyses of sample number according to MAP K-10 (A) and *M. tuberculosis* H37Rv (B) densities, estimated by TaqMan real-time PCR targeting MAP0865 (n=40 spleen, n=40 liver) or ext-RD9 (n=7 spleen, n=9 lung), respectively. All these accurate real-time PCR results are observed for a host DNA amount < 3 µg in a final volume of 25 µl, and DNA purity at 260/280 nm ratio = 1.89±0.08.

Using TaqMan real-time PCR targeting MAP0865, the *in vitro* limit of quantification (Figure 4A, on the left) was estimated at 1-10 genome equivalents (ge). When the same target was used to quantify DNA from the optimized tissue extraction method, and according to optimal DNA purity and optimal host DNA amount (detailed below), the limit of quantification was estimated at 100 ge/mg (2 log_10_ ge/mg) of spleen or liver (Figure 4A, on the right). In contrast, for *M. tuberculosis*, the *in vitro* limit of quantification was 100 - 1000 ge (i.e. 2 - 3 log_10_ ge: Figure 4B, on the left), corresponding to limits of quantification estimated at 1,000,000 ge/mg of lung (i.e. 6 log_10_ ge/mg: Figure 4B, on the right), or 10,000 ge/mg of spleen (i.e. 4 log_10_ ge/mg: Figure 4B, on the right). It must be also noticed that no more than 7 log_10_ ge/mg of MAP (Figure 4A, on the right) was detected in spleen and liver using MAP0865 target (Figure 4B, on the right); this did not reflect a detection ceiling, but rather the maximum number of MAP organisms that we observe after infection of C57BL/6 mice.

### PCR inhibition by host DNA

To overcome the limitation of bacterial detection, one could in theory use larger tissue samples. However, this may come at the expense of poorer quality total DNA and/or a greater quantity of host DNA. To test this, we investigated extractions from different organs, using 1 or 2 PCI step (n=384). As expected, the total extracted DNA increased proportionally according to organ weight (Figure S1), with a constant yield of ~ 3 log_10_ ng/mg of liver, ~ 4 log_10_ ng/mg of spleen and ~ 3.5 log_10_ ng/mg of lung (Figure S2). In parallel, DNA purity at 260/280 nm (Figure S1), and 260/230 nm (Figure S2), decreased according to the organ mass; 1 log_10_ ng of host DNA was lost from liver during the second step of PCI (Figure S1), with minimal gain in DNA quality, as observed with the 260 nm/280 nm (Figure S1) and 260/230 nm (Figure S2) ratios.

**Figure 5:**
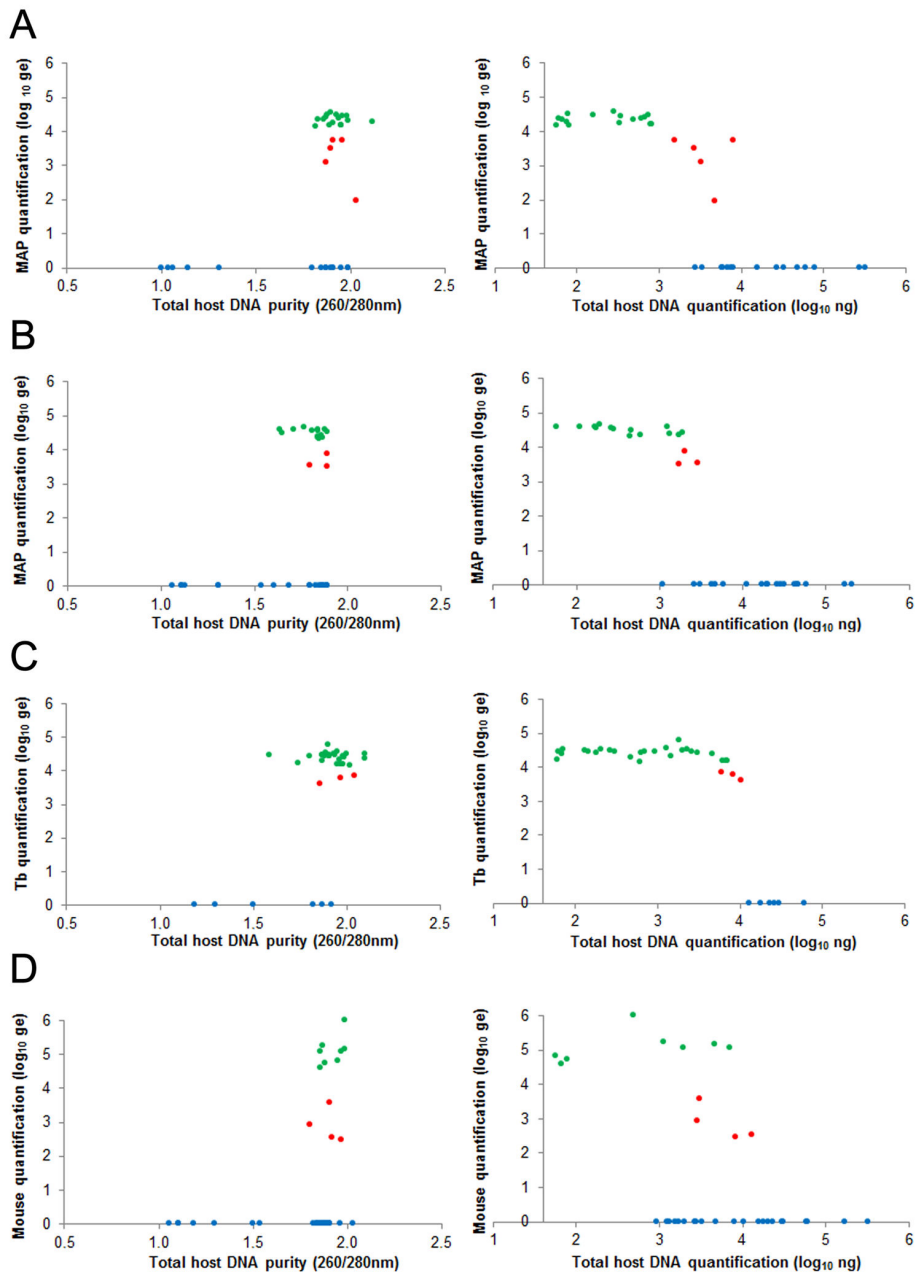
Accurate (green), inaccurate (red), and inhibited (blue) quantification of MAP-K10 in liver (A, n=36) and spleen (B, n=36) by TaqMan real-time PCR (MAP0865 gene), of *M. tuberculosis* H37Rv in lung (C, n=36) by TaqMan real-time PCR (ext-RD9 element), and of C57BL/6J mouse cells (D, n=36) by SyberGreen real-time PCR (PTGR2 gene), according to host DNA purity at 260/280 nm (on the left), host DNA quantity at 260 nm (on the right).

To determine whether host DNA could affect PCR-based detection of bacterial DNA, we selected 36 DNA samples presenting the complete spectrum of concentration and quality, then spiked these with a fixed amount of DNA from MAP (4.4±0.3 log ge/reaction) or *M. tuberculosis* (4.3±0.1 log ge/reaction). Three kinds of DNA samples were observed: 1) those where the real-time PCR estimated DNA density was similar to controls (i.e. no inhibition), 2) those where the real-time PCR estimated DNA density was lower than seen with controls (i.e. partial inhibition), and 3) those where there was no detection of DNA (i.e. complete inhibition). These three kinds of samples are graphically presented as accurately quantified (green points), inaccurately quantified (red points) or not quantified (blue points), as shown when MAP DNA was added to liver (Figure 5A), MAP DNA was added to spleen (Figure 5B) and *M. tuberculosis* DNA was added to lung (Figure 5C). We also tested the ability to detect host DNA in these same organs; indeed inhibition was also seen when targeting a host gene (Figure 5D). While accurately quantified DNA samples presented high purity (260/280 nm of 1.92±0.05 for the liver, 1.80±0.06 for the spleen and 1.91±0.08 for the lung), high DNA purity alone was not sufficient to achieve accurate mycobacterial quantification. Only samples with < 3 log_10_ ng of host DNA were accurately quantified, while samples with > 4 log_10_ ng of host DNA were not reliably quantified by real-time PCR.

## Conclusions

As a general principle, the reliability of a laboratory assay, such as PCR, is heavily dependent on the input material tested. Our data indicate that steps employed prior to PCR, namely sample processing and DNA extraction, affect the capacity to detect and quantify bacterial genomes inside tissue samples. Specifically, we showed that bead-beating was necessary for efficient disruption of tissue and that for the recovery of mycobacterial DNA, commercial spin column kits yielded 10-100-fold less DNA than the reference mycobacterial DNA extraction method. As different studies have reported highly variable findings when searching for mycobacteria drug resistance [30], or mycobacteria in diseases such as Buruli Ulcer [31], sarcoidosis [32], Blau syndrome [33] and Crohn’s disease [34], it may be that one source of this variability stems from technical differences between study protocols, *prior to* the conduct of the PCR reaction.

To overcome the limits of detection, a larger sample could in theory be processed and tested by PCR. Our data indicate that with larger tissue samples, the quality of DNA was decreased and the increased amount of host DNA led to partial, then complete inhibition of bacterial PCR. While we have not formally studied bacterial genera other than mycobacteria, we suspect that these findings pertain to the detection of other intracellular microorganisms in clinical samples, or other microorganisms in matrices, such as food, given that the inhibitory effect of excess DNA was documented even when amplifying a host target.

Mixed DNA is increasingly being used as the template for metagenomic studies that aim to overcome investigators’ specified interests and simply describe the complete microbial population present in a sample. Our data indicate that the kit used for the Human Microbiome Project is poorly suited for the detection of mycobacteria, even in the ideal scenario when they are present in pure culture. Compared to Gram positive and negative bacteria [35], mycobacteria present a particular challenge for DNA extraction because of their complex cell wall, comprised of a modified peptidoglycan covalently attached to an arabinogalactan layer, which in turn is linked to very long-chain fatty acids called mycolic acids [36]. However, the challenges with extracting mycobacterial DNA are not confined to this genus, we posit that agnostic studies that catalogue a sample by metagenomic methods may be subject to extraction bias, wherein certain organisms are less likely to be detected due to relative efficiency of DNA extraction. Further studies are indicated with other bacteria, in other types of samples, to optimize the DNA extraction step, as a prelude to targeted and agnostic downstream investigations aimed at determining the microbial contributors to health and disease.

## Supporting Information

Appendix S1 **Materials and methods for real-time PCR.**
(DOC)

Figure S1 **Total host DNA measured at 260 nm (on the left), and DNA purity measured at 260/280 nm (on the right), after DNA extraction from liver (A, n=32), spleen (B: n=32), and lung (C: n=32) of C57BL/6 mice, including 1 (in green) or 2 (in red) steps of phenol:chloroform:isoamyl (PCI) purifications.**
(TIF)

Figure S2 **Total host DNA measured at 260 nm (on the left) by organ weight, and DNA purity measured at 260/230 nm (on the right), after DNA extraction from liver (A, n=32), spleen (B: n=32), and lung (C: n=32) of C57BL/6 mice, including 1 (in green) or 2 (in red) steps of phenol:chloroform:isoamyl (PCI) purifications.**
(TIF)

Table S1 **Primers and probes used in this study.**
(DOC)
