# Supplementary material for: The Critical Role of DNA Extraction for Detection of Mycobacteria in Tissues
Source: PLoS One. 2013 Oct 23;8(10):e78749. doi: 10.1371/journal.pone.0078749 (PMC3806855; doi:10.1371/journal.pone.0078749)
Supplement: Appendix S1 — Materials and methods for real-time PCR. (DOC) [file pone.0078749.s001.doc]

Real-time PCR assays were done using a 7300 Real-Time PCR System (Applied Biosystems). The relative quantification of MAP K-10 DNA (genome of 4,829,781 bp corresponding to 5.30 fg per copy), *M. tuberculosis* H37Rv DNA (genome of 4,411,532 bp corresponding to 4.84 fg per copy) and C57BL/6 mice DNA from spleen (genome of 2,716,965,481 bp corresponding to 2.98 pg per copy) was performed with independent 10-fold serial dilutions of genome equivalents (ge) from 7 log_10_ copies until 0 copy in triplicate, and cycle Thresholds of 0.02 ΔRn and 0.1 ΔRn for SYBR Green and TaqMan assays respectively. All real-time PCR analyses included a negative process control (extracted DNA from non-infected mice) as well as a negative amplification control (reaction with water as template). SYBR Green real-time PCR assays (qPCR MasterMix 2X Maxima SYBR Green/ROX, Fermentas) were performed following manufacturer recommendations (final concentrations of 1X SyberGreen buffer, 0.3 µM of each primers, and 5 mM of MgCl_2_) in final volume of 25 µl (2 min at 50°C, 10 min at 95°C, 40 cycles of 15 s at 95°C, 30 s at annealing temperature, and 30 s at 72°C as extension and detection step, followed by a dissociation curve step). TaqMan real-time PCR assays (qPCR MasterMix 2X Maxima Probe, Fermentas) were performed according to manufacturer recommendations (final concentrations of 1X TaqMan buffer, 0.3 µM of each primers, 0.2 µM of probe, and 4 mM of MgCl_2_) in a final volume of 25 µl (2 min at 50°C, 10 min at 95°C, 40 cycles of 15 s at 95°C, 30 s at annealing temperature as elongation and detection step, and 30 s at 72°C). For MAP quantification, TaqMan probes were synthesized by Invitrogen with FAM as reporter on 5’ side, and MGBNFQ as quencher on 3’ side. For *M. tuberculosis* quantification, locked nucleic acid (LNA) probe was synthesized by Integrated DNA Technologies with JOE as reporter in order to avoid G quenching effect in 5’ side, and BHQ-1 as quencher on 3’ side.
