# Supplementary figures and images for: The Critical Role of DNA Extraction for Detection of Mycobacteria in Tissues
Source: PLoS One. 2013 Oct 23;8(10):e78749. doi: 10.1371/journal.pone.0078749 (PMC3806855; doi:10.1371/journal.pone.0078749)

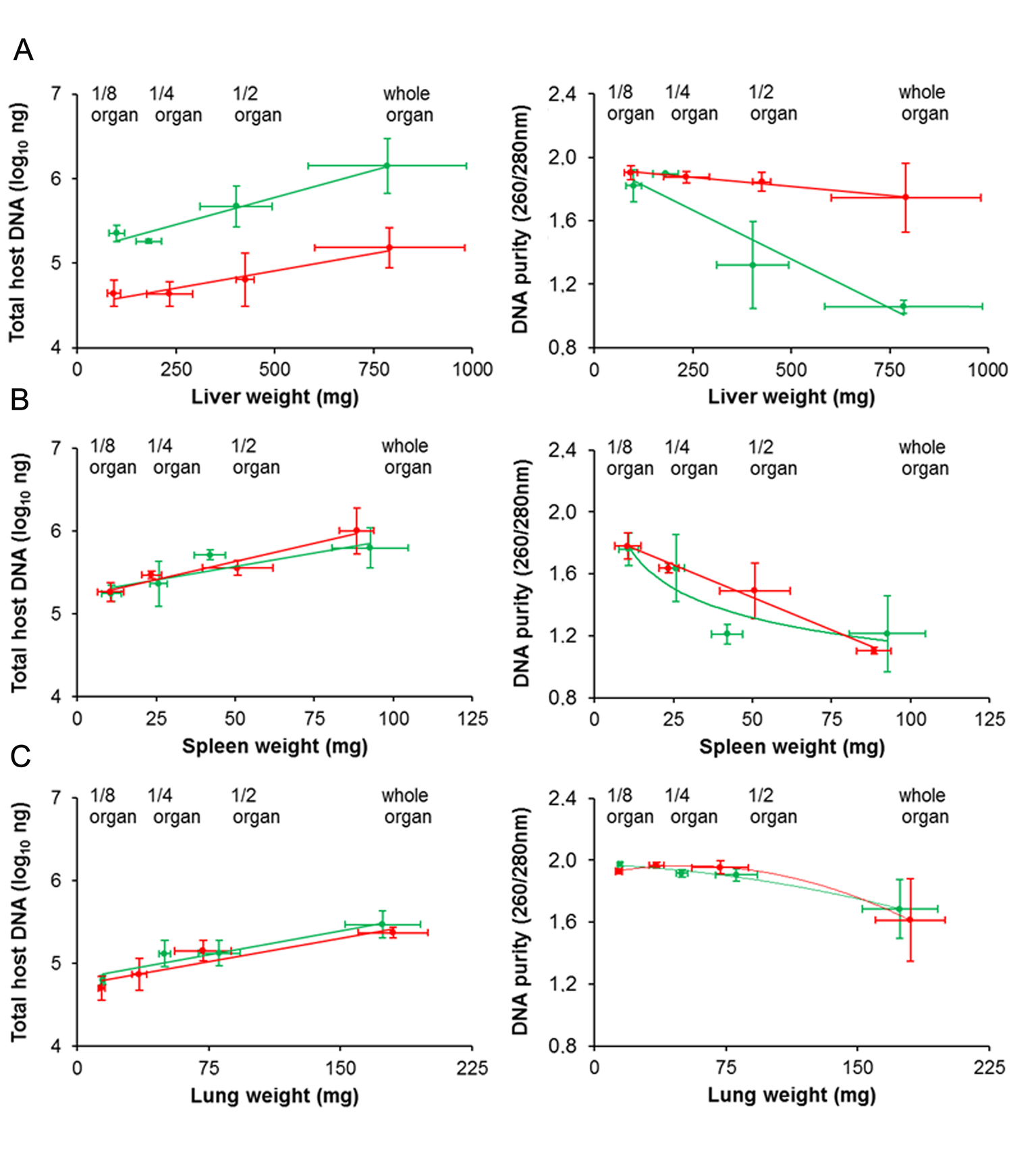

Supplement: Figure S1 — Total host DNA measured at 260 nm (on the left), and DNA purity measured at 260/280 nm (on the right), after DNA extraction from liver (A, n=32), spleen (B: n=32), and lung (C: n=32) of C57BL/6 mice, including 1 (in green) or 2 (in red) steps of phenol:chloroform:isoamyl (PCI) purifications. (TIF) [file pone.0078749.s002.tif]

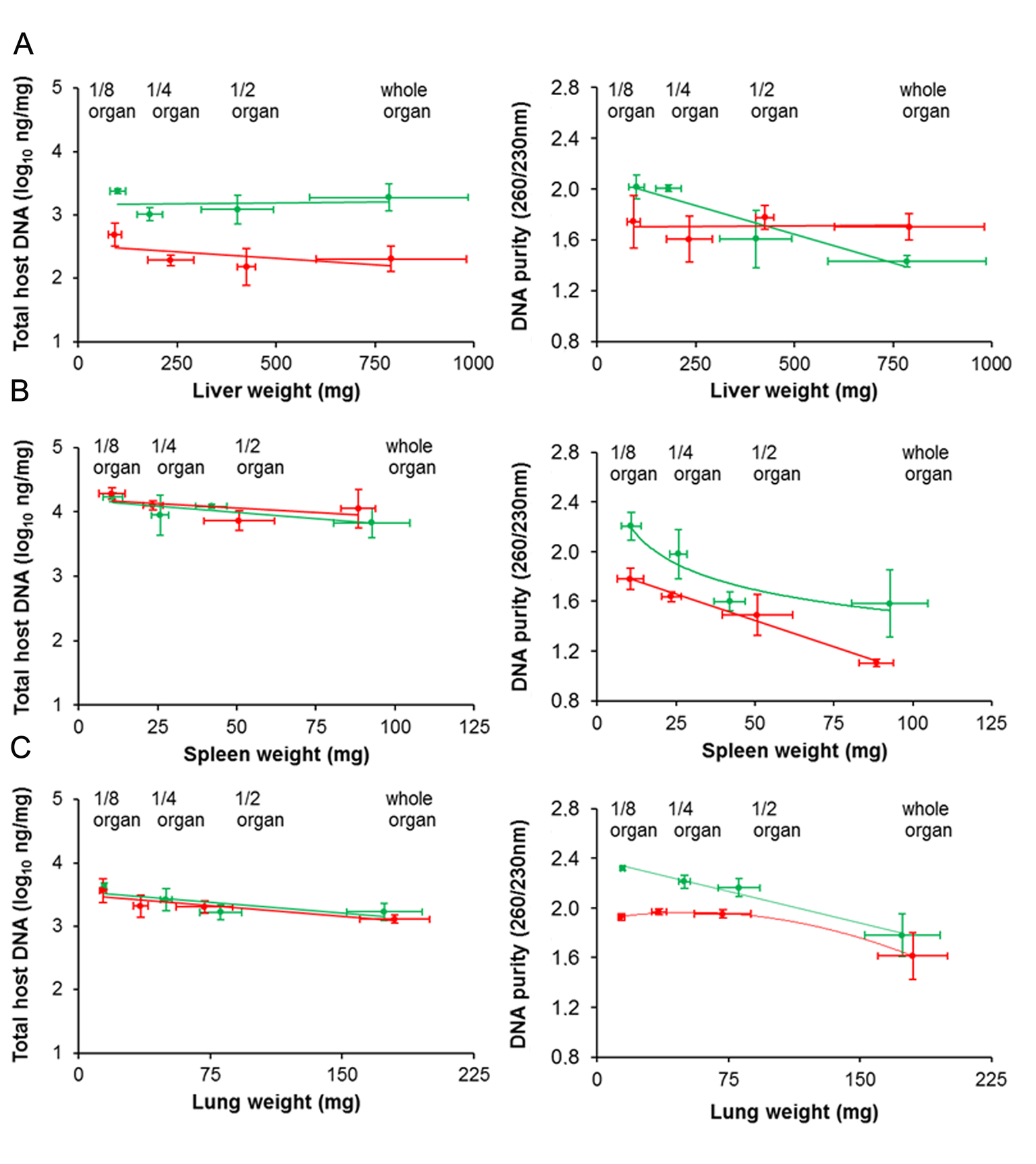

Supplement: Figure S2 — Total host DNA measured at 260 nm (on the left) by organ weight, and DNA purity measured at 260/230 nm (on the right), after DNA extraction from liver (A, n=32), spleen (B: n=32), and lung (C: n=32) of C57BL/6 mice, including 1 (in green) or 2 (in red) steps of phenol:chloroform:isoamyl (PCI) purifications. (TIF) [file pone.0078749.s003.tif]
