# Supplementary material for: The Critical Role of DNA Extraction for Detection of Mycobacteria in Tissues
Source: PLoS One. 2013 Oct 23;8(10):e78749. doi: 10.1371/journal.pone.0078749 (PMC3806855; doi:10.1371/journal.pone.0078749)
Supplement: Table S1 — Primers and probes used in this study. (DOC) [file pone.0078749.s004.doc]

| Targeted element | Sequence (5’-3’) ^a^ | Annealing  temperature (°C) | Reference ^b^ |
| --- | --- | --- | --- |
| MAP0865 | F: TACCGAATGTTGTTGTCACCG | 58 | Imirzalioglu, *et al*. |
| (MAP gene) | R: TGGCACAGACGACCATTCAA |  |  |
|  | P: CCGGTCCCAGGTGTGTTCGAGTTG |  |  |
| Ext-RD9 | F: GCCACCACCGACTCATAC | 60 | Halse, *et al*. |
| (MTC element) | R: CGAGGAGGTCATCCTGCTCTA |  |  |
|  | P: G+TT+CTTCAG+CTGGT+CC |  |  |
| PTGER2 | F: TACCTGCAGCTGTACGGCCAC | 60 | Alcoser, et al. |
| (eukaryotic gene) | R: GCCAGGAGAATGAGGTGGTC |  |  |

^a^ F stands for forward primer, R stands for reverse primer, P stands for probe, + stand for locked nucleic acid.

^b^ Imirzalioglu, *et al*. 2011, J. Clin. Microbiol. 49:1843-1852. Halse, *et al*. 2011. J. Clin. Microbiol. 49:2562-2567. Alcoser, *et al*. 2011, BMC Biotechnology 11:1-19.
